# The Effect of Age of Dam and Birth Rank on the Reproductive Performance of Ewes as One- and Two-Year-Olds

**DOI:** 10.3390/ani11030770

**Published:** 2021-03-10

**Authors:** Emma Pettigrew, Rebecca Hickson, Steve Morris, Paul Kenyon, Rene Corner-Thomas, Emmanuelle Haslin, Hugh Blair

**Affiliations:** School of Agriculture and Environment, Massey University, Palmerston North 4412, New Zealand; R.Hickson@massey.ac.nz (R.H.); S.T.Morris@massey.ac.nz (S.M.); P.R.Kenyon@massey.ac.nz (P.K.); R.Corner@massey.ac.nz (R.C.-T.); E.Haslin@massey.ac.nz (E.H.)

**Keywords:** ewe lamb, breeding, reproduction, mature ewe, dam age, birth rank

## Abstract

**Simple Summary:**

Lambs that are born to ewe lambs are not commonly selected as replacement ewes for sheep flocks in New Zealand, as they are born smaller and later than lambs born to mature ewes, hindering their chances of being heavy enough to breed as ewe lambs themselves. By selecting lambs born to ewe lambs, farmers can utilize animals that are available, and potentially increase genetic gain and ewe production efficiency. This experiment found that lambs born to ewe lambs are lighter than those born to mature ewes until two years of age, but have similar body condition scores, and rates of lamb production, if they are heavy enough to be bred as ewe lambs themselves. Therefore, farmers could select lambs born to ewe lambs that are heavy enough to achieve breeding targets.

**Abstract:**

Currently, 30–43% of New Zealand sheep farmers breed their ewe lambs, but few retain the offspring as replacements for their flock. No difference in lamb production as a yearling among singletons and twins born to ewe lambs and twins born to mature ewes has been reported, provided the ewe lambs had reached the 60–65% of their likely mature weight prior to breeding at seven to eight months of age. The aim of this experiment was to determine the lamb production from singletons and twins born to ewe lambs and twins born to mature ewes during their first two years of lambing. The experiment included 8-month-old ewes born as twins to mature ewes (M2, *n* = 135), singletons born to ewe lambs (L1, *n* = 135), and twins born to ewe lambs (L2, *n* = 88), bred during the same period to the same rams, over two years. The efficiency of lamb production (total litter weight at weaning divided by the pre-breeding weight of the ewe, for all ewes presented for breeding) after two years of production was not significantly different (*p* > 0.05) among the groups (0.40 ± 0.02, 0.39 ± 0.02, and 0.39 ± 0.03, for M2, L1, and L2, respectively).

## 1. Introduction

Currently, 30–43% of farmers breed their ewe lambs (young ewes aged up to 15 months of age) at eight to nine months of age [1], allowing them to lamb for the first time at 12 months of age. By doing so, increases in the number of lambs produced per ewe per lifetime and in the number of lambs produced on farm each year can be achieved [1,2]. If lambs born to ewe lambs are selected as replacement females to enter the breeding flock, there is the potential to increase the rate of genetic gain, by decreasing the generation interval of the population [1,3]. Commonly in New Zealand, progeny born to ewe lambs are slaughtered for meat production and are not selected as replacements, negating the potential advantage. Therefore, there is currently very little information in the literature on the maternal performance of replacements born to ewe lambs over their productive life [4,5].

Lambs born to ewe lambs on New Zealand farms are usually born a month later than those born to mature ewes [1], yet must still reach developmental milestones at the same calendar date as lambs born to mature ewes to fit within the seasonal lambing system. Furthermore, they are lighter at birth and weaning [6,7]. Lambs that are born as twins, regardless of dam age, are also lighter than lambs born as singletons at both birth and weaning [1,8,9,10], and this difference in live weight persisted until at least eight years of age [11]. It is well documented that live weight at joining has a positive influence on reproductive performance for both mature ewes and ewe lambs [12,13,14,15]. Therefore, it would be expected that lambs born to ewe lambs, especially those born as twins, may have greater difficulty achieving suitable live weights for breeding at seven to nine months of age and, therefore, would display poorer reproductive performance.

It has been reported that lambs born as singletons or twins to ewe lambs were lighter at birth and to 12 months of age, and until the weaning of their first lambs at 26 months of age [4], compared to singletons or twins born to mature ewes. The same authors also reported that singletons born to ewe lambs were lighter than singletons born to mature ewes for the first 12 months of their life, but then had similar live weights until three years of age [7] and four years of age [5]. All of these studies reported there were no differences in the number or weight of lambs produced at birth [5] and weaning [4,5] among the respective comparisons. Furthermore, ewes born to ewe lambs produced lambs of greater weights at birth [4,7] and weaning [7]. There were also no differences in the body condition scores of the ewes among the respective groups [4,5,7,16]. These studies had limited numbers of ewes in each group, ranging from 17 to 41, and did not breed their ewes as ewe lambs, so further experiments with larger population sizes and trial designs are warranted.

The aim of this experiment was to determine whether being born as a singleton or twin to a ewe lamb compared with being born as a twin to a mature ewe caused differences in live weight, body condition score, and maternal performance (number of lambs produced and weight of lambs produced) for the first two years of life, when bred first at seven months of age.

## 2. Materials and Methods

This study was conducted at Massey University’s Riverside farm (latitude 40°84′ S, longitude 175°62′ E) 11 km north of Masterton, and Keeble farm (latitude 40°40′ S, longitude 175°60′ E) 5 km south of Palmerston North, New Zealand. The experiment ran from January 2018 to December 2019 with the approval of the Massey University Animal Ethics Committee (MUAEC 17/16).

### 2.1. Background

Three hundred and fifty eight Romney ewe lambs born during October to November 2017, to either ewe lambs, as either singletons (L1; *n* = 135) or twins (L2; *n* = 88), or to mature ewes, as twins (M2; *n* = 135) were selected for this experiment [10]. Singletons born to mature ewes were not considered as they are not usually retained as replacements and there is no productive advantage of doing so. This paper reports on the ewe lambs from their weaning (D0, 3rd January 2018, average age 82 days) to the weaning of their second lambs (D714). The M2 ewe lambs were selected from a larger population (*n* = 647; [10]), and included 135 (selected randomly) of the heaviest 270 lambs at weaning, the L1 ewe lambs included the heaviest 135 of 184 lambs, whereas L2 ewe lambs included the entire population of female lambs present at weaning. The ewes were managed at Riverside farm from their birth (September–November 2017, [10]), and were relocated to Keeble farm in January 2019 (D392), where they remained for the duration of the experiment.

Prior to the first breeding period (May 2018), the goal was to ensure as many ewe lambs as possible were heavy enough for breeding. The current industry recommendations in New Zealand include ewe lambs needing to be a minimum of 40 kg or 65% of their mature weight prior to breeding [17]. However, there are indications that ewes born to ewe lambs are lighter than those born to mature ewes from birth to weaning and until at least eight years of life [10]. Therefore, for the present study a minimum live weight of 39 kg for breeding as ewe lambs was used. Therefore, the nutritional management of the treatment groups needed to differ, from their weaning (average weights of 29.6, 26.0, and 21.5 kg, for M2, L1, and L2 ewe lambs selected, respectively) until breeding, because lambs born to ewe lambs were much lighter at weaning. Further, the farm that the ewe lambs were on was exposed to drought conditions through the autumn (February–April 2018), and therefore the use of supplements was required to achieve target growth rates for L1 and L2 ewe lambs. This approach was taken as part of normal farm practice to ensure that as many light ewe lambs as possible reached target breeding weights.

### 2.2. Nutritional Management

The ewe lambs were managed together from 13 days (D13) after weaning from their mothers (3 January 2018; D0) until D76, at which time the M2 lambs were grazed separately with no supplement until D127. Ewe lambs grazed various pastures and up to 240 g/lamb/day of commercial supplement provided, and after D144 all ewe lambs received only ryegrass-based pasture. 

At introduction of the ram at D127 (10 May 2018), all ewe lambs heavier than 39 kg (*n* = 135, 95, and 29 for M2, L1, and L2 ewe lambs, respectively) were merged into one group, whilst ewe lambs lighter than 39 kg (*n* = 0, 40, and 59 for M2, L1, and L2 ewe lambs, respectively) were grazed in a separate group without rams. At D144, ewe lambs that were not with the ram were weighed again, and a further 12 L1 and seven L2 ewe lambs were added to the group with the rams.

At D161 (end of the breeding period), all ewe lambs were grazed together until D265. At D265, all pregnant ewe lambs (*n* = 66, 57, and 24 for M2, L1, and L2 ewe lambs, respectively) were paddocked for lambing, separated based on first or second 17-day period, then randomly allocated over two sets of four paddocks. Nonpregnant ewe lambs (*n* = 212) were grazed in a separate group. Lambing occurred between D265 and D308. From D308 until D379, the ewe lambs that had lambed were rotationally grazed in two groups, based on lambing in the first or second 17-day period.

The 16-month-old ewes had their first lambs weaned on D379. From D379, all ewes were managed as one group. Due to drought conditions, the ewes were supplemented with grass baleage and commercial concentrate feeds during their second autumn. 

At D615, 13 days before the planned start of lambing, the ewes were allocated to their paddocks for the lambing period. Ewes identified as not pregnant at pregnancy detection (*n* = 17) were removed, and grazed separately to pregnant ewes. Pregnant ewes were separated based on expected lambing date (determined by crayon marks from harnessed rams at mating) and balanced to achieve a spread of single and twin pregnancies in each paddock. All triplet-bearing ewes (*n* = 9) were placed in one paddock alongside single and twin-bearing ewes. At D660, lambing groups (and the nonpregnant ewes) were merged to create two mobs, and rotationally grazed in these two groups until D714.

### 2.3. Ewe Live Weight and Body Condition Score Measures

The ewe lambs were weighed within two hours off pasture at 34 time points throughout the experiment. Body condition scores (BCSs; [18]; scale 1–5, 1 = emaciated, 5 = obese) of the ewes were also recorded at 16 of these events.

### 2.4. Reproductive Measures

At D59 (3 March 2018), the ewe lambs were joined with crayon-harnessed vasectomised rams (ratio of approximately 1:50) for 68 days (D59–D127). At the end of each 17-day period (D76, D93, D110, and D127), crayon marks were recorded on all ewe lambs, to detect if a ewe lamb was mounted/crayon marked, as an indicator of oestrus and puberty attainment (Allison et al., 1975) and crayon colours were changed. Crayon marks on the ewe lamb rumps were scored using a 0–3 score, indicating the incidences of being mounted by the ram (0 = no marks, 1 = one mark, 2 = two marks, 3 = three or more marks [19]). Ewe lambs were considered to have shown oestrus as an indicator of puberty if they had a crayon mark score on their rump of two or three at any time crayon marks were recorded [20]. At D76, L1 and L2 ewe lambs were separated from M2, to allow preferential feeding for the L1 and L2 ewe lambs (see earlier details), along with three vasectomised teasers (ratio of approximately 1:45), with L1 and L2 ewe lambs remaining as a second group with the remaining teaser rams (ratio of approximately 1:53). On D127 (10 May 2018), the vasectomised rams were removed.

At D127 (10 May 2018, average age of 209 days), all ewe lambs that were 39 kg or heavier (*n* = 258) were joined as one group with entire, harnessed Romney rams for 34 days (two 17-day reproductive periods), at a ratio of approximately 1:39 (*n* = 135, 95, and 29 M2, L1, and L2 ewe lambs, respectively). At the end of each reproductive cycle (17 days; D144 and D161), crayon marks were recorded on ewe lambs’ rumps. Crayon colours were changed on D144. At D144, any ewe lambs that were found to be 39 kg or above and not already in the breeding group (*n* = 0, 12, and 7 for M2, L1, and L2 ewe lambs, respectively) joined with the breeding group. One additional ram was also added, creating a ratio of approximately 1:38 of rams to ewe lambs. At D161, crayon marks on the rump were recorded, and the rams were removed from the ewe lambs. Only 81 ewe lambs (*n* = 0, 29 and 52 for the M2, L1, and L2 ewe lambs, respectively) of the total 358 were not presented for breeding, due to being too light.

Pregnancy detection occurred at D211 (2 August 2018) and D568 (25 July 2019), via transabdominal ultrasound, to detect the number of foetuses. The breeding period of conception was defined using crayon marks, for all ewe lambs diagnosed as pregnant, to allocate paddocks for lambing. Pregnant ewes were identified as becoming pregnant during the first 17-day period of breeding if they had only the first crayon colour on their rumps (*n* = 254). Those pregnant and showing both or only the second crayon colour on their rumps were identified as becoming pregnant in the second 17-day period of breeding (*n* = 25). Pregnant ewes that had no crayon marks at the end of the breeding period (*n* = 28) were also assumed to be pregnant during the second 17-day breeding period.

At D481 (29 April 2019), ewes were joined with entire, crayon-harnessed Romney rams at a ratio of approximately 1:59 for 34 days (two 17-day reproductive cycles; rams removed at D515). The crayon harness colours were changed on d498 to detect if a ewe was mounted during either 17-day breeding period. On D498 and D518, crayon marks on the ewes’ rump were scored.

### 2.5. Management and Data Collection during Lambing Periods

During the lambing period in 2018 (D265–D308), ewe lambs were checked twice daily at approximately 9 a.m. and 3 p.m., and in 2019 (D621–D663) the ewes were monitored twice daily at approximately 8:30 a.m. and 3 p.m., by one of two trained operators. Lambs that were old and strong enough to stand were tagged and identified to their dam, and if any lambs were not yet standing, the entire litter was left until the next check. The sex of the lamb(s), its birth rank, date and paddock of birth, and birth weight were recorded. Maternal behaviour scores (1–5 score; [21]) were recorded for all dams that had a live lamb at tagging. Any dead lambs were weighed and recorded. Lambs that were dead at the time of tagging were considered born dead, and dead lambs that were collected after tagging, until 3 days after the end of the lambing period (2018: D311; 2019 D666), were considered to have died between birth and the end of the lambing period. At D308 and D660 (average of day 24 of lactation in both years; L24), all lambs were weighed, and the tail removed using a hot iron (docked), and all male lambs were castrated using a rubber ring. In 2019, there were five lambs born on or after the docking date that had all birth measurements taken and were additionally tail docked and castrated (if male) using a rubber ring when tagged. At D379 and D714 (average of days 95 and 78, respectively, of lactation), lambs were weaned and weighed. Lambs that were not present at weaning were considered to have died between tagging and weaning.

### 2.6. Ewe Health Issues in 2019

At D398 and D442, ewes were dosed with zinc capsules (Time Capsule^®^ Adult Sheep, AgriTrade, Hamilton, New Zealand) to provide protection against facial eczema. At D450, D498, and D567, faecal samples were taken to determine faecal egg counts for drench management. Ewes were drenched with Genesis Ultra Hi Min (Boehringer-Ingelheim, Auckland, New Zealand) sheep drench at D450, D503, and D597. Ewes were crutched (removal of the wool from the breech and belly area of the fleece) at D484. During the duration of the experiment there were 16 (M2 *n* = 3, L1 *n* = 8, and L2 *n* = 5) ewe deaths that occurred prior to the lambing period, and nine (M2 *n* = 5, L1 *n* = 2, and L2 *n* = 2) that occurred during the lambing period. These death rates (M2: 6%, L1: 8%, L2: 8%) were within the normal ranges (2.8–20% per annum) for New Zealand ewes [22].

### 2.7. Data Handling for 2018

The trait of percentage of ewe lambs showing oestrus as ewe lambs (and therefore assumed to have achieved puberty) prior to the introduction of the ram (Oestrous %) was defined based on the presence or absence of crayon marks from the vasectomised rams. Ewe lambs with crayon scores of 2–3 while joined with the vasectomised rams were considered to have shown oestrous, an indicator of puberty attainment. The number of ewe lambs presented for breeding in both reproductive cycles of breeding, or just the second reproductive cycle of breeding, or not presented for breeding were recorded. Based on these results, those that were presented for both reproductive cycles, and only for the second reproductive cycle were considered presented for breeding. The entire population included all ewe lambs that either were or were not presented for breeding for each treatment group. The number of ewe lambs that were pregnant, was considered firstly as a percentage of all ewe lambs presented for breeding, and secondly for the entire population, within each treatment group. The percentage of ewes that were multiple bearing at the time of pregnancy detection was based on only those that were diagnosed as pregnant at pregnancy detection for each treatment group. The percentage of ewe lambs pregnant during the first cycle of breeding was determined retrospectively, using the date of lambing, and considered only ewe lambs that were presented for breeding during both reproductive cycles of breeding. The number of lambs present at birth (NLB) and weaning (NLW) were determined as percentages, for the ewe lambs that were presented for breeding and the total population for each treatment group.

Lamb survival to tagging was determined based on whether a lamb was alive or dead at the time of tagging. Lamb survival to weaning was determined on the weaning weight measurements, with lambs that were not present at weaning considered to have died between tagging and weaning.

### 2.8. Statistical Analysis

Statistical analysis was carried out using SAS version 9.4 software (SAS Institute, Cary, NC, USA). Live weights of ewes were analysed with a linear mixed model, with the fixed effects of treatment group, day of measurement, pregnancy rank (zero, one, two, or three foetuses) in 2018 and 2019, and the interaction of treatment group and day of measurement. The random effect of animal was included to account for repeated measures. This selection among only the heaviest (42%) of lambs caused skewness and kurtosis in live weights of the M2 ewe lambs in 2018. However, the skewness and kurtosis were considered insufficient to adversely affect ANOVA tests of significance because the ratio of population sizes was equal to 150% and, therefore, ANOVA without transformation of the data was conducted. A univariate analysis was carried out for live weights on each day of measurement among the three groups, as a test for normality using the Kolmogorov–Smirnov method. A general linear model was used with a Levene’s test for homogeneity of variance for live weights within each day of measurement among the three groups.

Body condition score was analysed with a generalised linear model, assuming a Poisson distribution, with fixed effects of treatment group, day of measurement, and pregnancy rank (zero, one, two, or three foetuses) in 2019, and the interaction of treatment group and day of measurement. The pregnancy rank in 2018 was initially considered, but was not significant (*p* > 0.05), and so was removed from the model. Animal was included as a random effect to account for repeated measures.

The data of ewe lambs that were heavy enough to be bred for either reproductive cycle and the probability of pregnancy occurring, based on pregnancy diagnosis results using live weights at the introduction of the ram as the independent variable, were analysed using logistic regression. A generalised linear model was used to analyse reproductive traits, based on a binomial distribution, and using the fixed effect of treatment group.

The number of lambs present at birth and weaning per ewe bred and per ewe of the entire population were analysed using a generalised linear model, assuming a Poisson distribution, with the fixed effect of treatment group.

Lamb birth weight (BWT), mid-lactation weight (L24; day 24 of lactation for 2018 and 2019), and weaning weight (WWT; days 95 and 78 of lactation for 2018 and 2019, respectively) were analysed using a mixed linear model, with the fixed effects of treatment group, sex of lamb, and birth rank of the lamb. Date of birth was added as a covariate, and the random effect of dam was included to account for repeated measures of twin or triplet lambs. The interaction of the treatment group and birth rank of the lamb was initially tested but was not significant (*p* > 0.05) and so was removed. Sex of lamb was not significant (*p* > 0.05) for mid-lactation and weaning weight in 2018, and so was removed from these models. Lambing paddock was considered for all models but was not significant (*p* > 0.05) and was removed.

Lamb survival at the time of tagging (used as an indicator of survival at birth) and weaning was analysed using a generalised linear model, assuming a binomial distribution, and included the fixed effects of treatment group and lamb birth rank. Sex of lamb was also included as a fixed effect in the models for both years but was not significant (*p* > 0.05) in 2018 analyses, so was removed from those models. Confidence intervals at the 95% level were used to test for differences in lamb survival to weaning for the treatment groups of dam age and lamb birth rank. The interaction of treatment group and birth rank of the lamb and the fixed effect of lambing paddock were initially tested but were not significant (*p* > 0.05) and so were removed from the model. Ewe maternal behaviour scores were analysed using a generalised linear model, assuming a Poisson distribution, and had the fixed effect of treatment group. In 2018, the fixed effects of birth rank, operator, and lambing paddock were also tested, but were not significant (*p* > 0.05) and so were removed from the model. In 2019, the fixed effect of birth rank was included in the model, and operator, and lambing paddock were also tested, but were not significant (*p* > 0.05) and so were removed from the model.

For both lambing periods, the average number of lambs weaned per ewe bred was analysed using a generalised linear model, assuming a Poisson distribution, with the fixed effects of treatment group and year. The random effect of animal was included to account for repeated measures. The average ewe efficiency was calculated as the total litter weaning weight of lambs divided by the breeding weight of the ewe, within each year of production. Ewes that were not presented for breeding as ewe lambs did not have values calculated for their first year but did have an efficiency calculation for their second year. The average litter weight at weaning per ewe bred and the average ewe efficiency were analysed using a mixed linear model with the fixed effects of treatment group and year. The random effect of animal was included to account for repeated measures for ewes that were presented for breeding in both years.

## 3. Results

### 3.1. Ewe Live Weights

**Figure 1:**
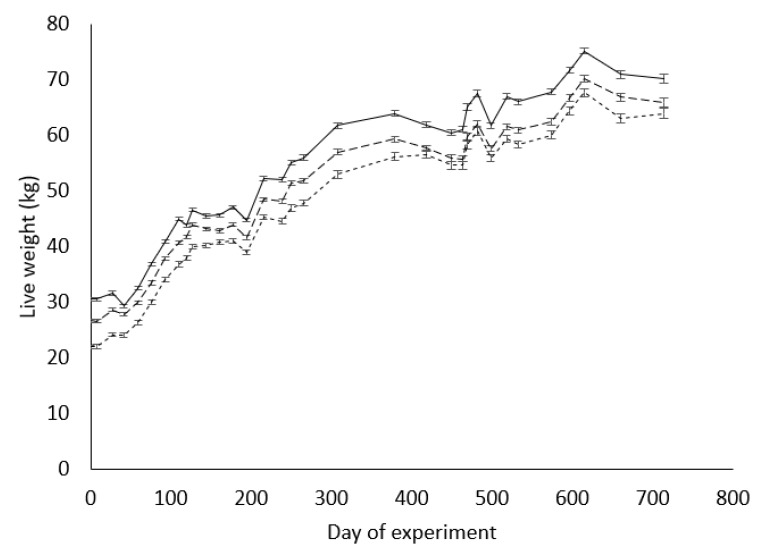
Least-squares means (error bars of ± S.E.M.) of live weight (kg) of ewes born to mature ewes as twins (M2; ―), or born to ewe lambs as singletons (L1; – –) or as twins (L2; ---) from their weaning (D0) to the weaning of their second lambs (D714).

Throughout the experiment, the M2 ewes were consistently heavier (*p* < 0.0001) than both other ewe groups from their own weaning (D0) until the weaning of their second lambs (D714; Figure 1). The L1 ewes were heavier (*p* < 0.0001) than the L2 ewes throughout the experiment, except on days 418, 449, 463, 469, 481, and 714, when there was no significant difference (*p* > 0.05) between the two groups.

### 3.2. Ewe Body Condition Score

**Figure 2:**
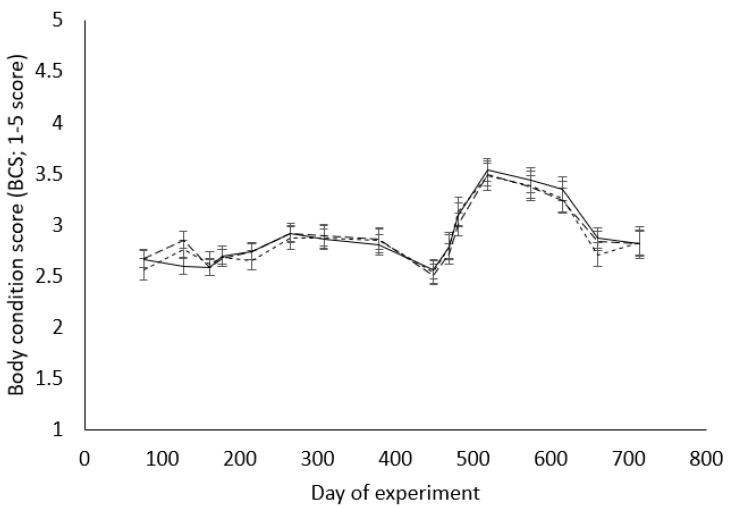
Body condition scores (BCSs; 1–5 score) of ewes born to mature ewes as twins (M2; ―), or born to ewe lambs as singletons (L1; – –) or as twins (L2; ---), from D76 after their weaning (D0) to the weaning of their second lambs (D714). Values are back-transformed LS means and error bars indicate 95% confidence intervals.

Throughout the experiment, there was no significant difference (*p* > 0.05) among the ewe groups for body condition scores, except for d127 when the M2 ewes had lower (*p* < 0.05) body condition scores than both the L1 and L2 groups, and day 660 when the L2 group had lower (*p* < 0.05) body condition scores than the M2 group (Figure 2).

### 3.3. Ewe Lamb Puberty

Fewer (*p* < 0.01) L2 than M2 ewe lambs reached puberty prior to the introduction of the ram, while L1 ewe lambs did not differ from either the M2 or L2 group (Table 1). In addition, fewer (*p* < 0.05) L2 ewe lambs were presented for breeding in the first and second 17-day periods of breeding than L1 ewe lambs. All M2 ewe lambs were presented for breeding in the first breeding period.

### 3.4. Ewe Lamb Probability of Pregnancy

**Figure 3:**
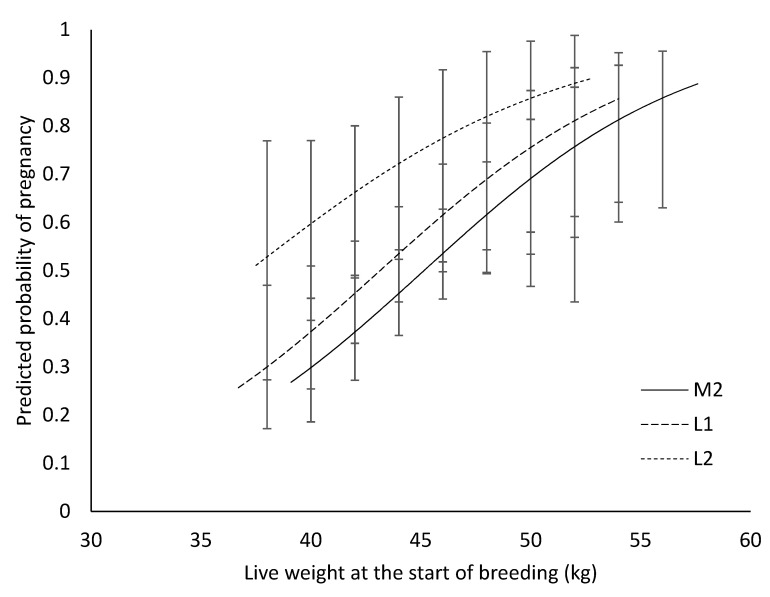
The effect of live weight (kg) at the start of breeding on the probability of pregnancy occurring over the breeding period, for the ewe lambs born to mature ewes as twins (M2; ―), or born to ewe lambs as singletons (L1; – –) or as twins (L2; ---) during their first breeding. Values are back-transformed LS means and error bars are 95% confidence intervals.

At the time of introduction of entire rams (D127) and at a given live weight, there was no difference (*p* > 0.05) among treatment groups in the percentage of ewe lambs likely to become pregnant (Figure 3). As live weight at the introduction of the ram increased, so did the likelihood of the ewe lamb conceiving during the breeding period, irrespective of treatment group.

### 3.5. Reproductive Performance

In 2018, there was no difference (*p* > 0.05) in the proportion of ewe lambs pregnant from each treatment group, when analysed based only on ewe lambs that were presented for breeding (either both breeding periods, or just the second breeding period; Table 2). However, when analysed based on the entire population, there were fewer (*p* < 0.01) L2 ewe lambs pregnant than both L1 and M2 ewe lambs. There was no difference (*p* > 0.05) in the proportion of pregnant ewe lambs bearing multiples or those pregnant in the first breeding period of those that were presented for the entire breeding period among the treatment groups (data not shown).

Of the ewe lambs presented for breeding in 2018 (either both breeding periods or just the second breeding period), there was no difference (*p* > 0.05) in the number of lambs weaned per treatment group (Table 2). Of the entire population, M2 and L1 ewe lambs weaned more (*p* < 0.02) lambs than L2 ewe lambs.

In 2019, there was no difference (*p* > 0.05) in the percentage of ewes that were pregnant, or the number of lambs present at pregnancy detection or weaning (Table 2) among the ewe treatment groups.

### 3.6. Progeny Live Weight

In 2018 and 2019, there was no interaction (*p* > 0.05) between treatment group (M2, L1, or L2) and progeny birth rank (singleton or twin) regarding the weight of progeny at birth, L24, or weaning. In 2018, there was no difference (*p* > 0.05) in the birth weight, weight at 24 days of lactation, or weaning weight of progeny born to M2, L1 and L2 ewe lambs (Table 3). In 2019, at birth, lambs born to M2 ewes were 0.3 kg heavier (*p* < 0.05) than lambs born to L1 and L2 ewes, which were not different (*p* > 0.05; Table 3). There was no difference (*p* > 0.05) in the weight of lambs at 24 days of lactation, and at weaning among the three treatment groups (Table 3). In 2018, at all ages, progeny born as singletons were heavier (*p* < 0.0001) than progeny born as twins (Table 3). In 2019, lambs born as twins did not differ in weight (*p* > 0.05) at birth to lambs born as triplets, but twins were heavier (*p* < 0.0001) than triplets at L24 and weaning.

### 3.7. Progeny Survival

In 2018 and 2019, there was no interaction (*p* > 0.05) between treatment group (M2, L1, or L2) and progeny birth rank (singleton or twin) on the survival of progeny at tagging, or to weaning. There was no difference (*p* > 0.05) in the survival of progeny at tagging, or to weaning of progeny born to M2, L1 and L2 ewe lambs, or between progeny birth ranks (singleton vs. twin; Table 4).

### 3.8. Ewe Maternal Behaviour Score at Lambing

In 2018 and 2019, there was no interaction among treatment groups (M2, L1, and L2) and progeny birth rank (singleton or twin; *p* > 0.05) for maternal behaviour scores (data not shown), and no difference (*p* > 0.05) among treatment group (2018 MBS 2.7 (95% CI: 2.2–3.2), 2.6 (2.1–3.2), and 2.4 (1.8–3.3), 2019 MBS 2.89 (2.45–3.41), 2.78 (2.33–3.27), and 2.53 (2.07–3.09) for M2, L1, and L2 ewe lambs, respectively) or progeny birth rank for maternal behaviour scores.

### 3.9. Ewe Average Production and Efficiency at Two Years of Age

Average lamb production for the first two years of production was measured per ewe bred within each year and is presented in Table 5. The average number of lambs weaned per ewe bred, average litter weight at weaning per ewe bred, and average efficiency (ratio of total progeny litter weight to ewe breeding weight per year, for all ewes presented for breeding) were not different (*p* > 0.05) among the treatment groups.

## 4. Discussion

The aim of this experiment was to determine whether being born as a singleton or twin to a ewe lamb or as a twin to a mature ewe (acting as a control group) caused differences in live weight, body condition score, and reproductive performance for the first two years of life, when bred first at seven-months-old. It was hypothesised that there would be no differences in lamb production among the ewe groups, but their live weights would be significantly different.

One limitation of this experiment was being able to select the M2 group of ewe lambs based on their weaning weights, while having to include all female progeny available from the L1 and L2 groups to have enough numbers. This situation is likely to reflect that seen commercially; however, where the population of lambs born to ewe lambs are fewer in number and lighter in weight at weaning. Therefore, some of the L1 and L2 ewe lambs were not heavy enough to be presented for breeding for their first breeding in 2018. At all measurements, M2 ewe lambs were heavier than L1 ewes, which were heavier than L2 ewes. In the literature, ewes born to ewe lambs were lighter than ewes born to mature ewes, until at least 2.5 years of age [4]. Interestingly, there were few differences in body condition scores among treatment groups throughout the experimental period. This indicates that at any given live weight, the L2 ewe lambs possibly had greater BCSs than the L1 ewe lambs, which were also possibly greater than the M2 ewe lambs.

In 2018, due to only a proportion of the L1 and L2 populations being heavy enough to be bred, the reproductive analysis was carried in two ways: performance per ewe presented for breeding, and performance per ewe of the population for each treatment group. When considering the entire population, there were fewer lambs produced by the two groups born to ewe lambs, especially those born as twins. This is due to the proportions of each group that were not presented for breeding, and, therefore, not given the opportunity to produce a lamb. When considering just the populations that were presented for breeding only, there were no differences in the number or weights of lambs weaned among the three groups, or rates of lamb survival, suggesting there is no consequence of breeding ewe lambs that were born to ewe lambs if they are heavy enough to be bred. There was no difference in the number of lambs born or weaned between ewes born as singletons or twins, to mature ewes or ewe lambs, during their first lambing at two years of age [4].

In 2018, the lambing percentage (number of lambs weaned per ewe presented for breeding) ranged between 45.2 and 52.8% among the three groups, which is lower than the national average of 65.4% for ewe lambs in 2018 [17], but within the range of 36.0–69.0% [1]. The ewe lambs in the present study were born in October, making them 7-months-old at breeding. This is a month younger than replacements in the industry, which are generally born to mature ewes in September. This likely explains why they are less likely to have attained puberty prior to breeding, resulting in a reduced reproductive performance [23]. The ewe lambs in this study were also bred at a minimum of 39 kg, while Beef and Lamb New Zealand recommend a minimum of 40 kg [17]. Removing the ewe lambs that were less than 40 kg at the start of the breeding period only increased lambing percentage by 2%, which does not explain the difference between the current results and the industry average. The percentage of each group showing oestrous activity prior to breeding was significantly lower than that previously reported [24]. The ewe lambs were on average 207-days-old at the start of breeding, while it was reported that ewe lambs first achieved oestrous at an average of 260–264-days-old [24]. The lower performance of these ewes is, therefore, possibly due to them being younger than traditional replacements at breeding, and more likely to reach puberty during, rather than before, the breeding period. The largest impact farmers could make to ensure that lambs born to ewe lambs will be suitable for breeding as ewe lambs themselves is to ensure that they are heavy enough to be bred.

In an average New Zealand flock of 3000 breeding ewes, where ewe lambs are bred, with a 30% replacement rate [22], there would be 900 ewe lambs presented for breeding. Assuming they have a 65% weaning rate from the ewe lambs presented for breeding [17], and half of the offspring were female, this leaves 293 ewe lambs born to ewe lambs to select from. If farmers were to select the top 55% of these ewe lambs based on weaning weight, that would mean 161 replacements would be born to ewe lambs, while the other 739 of the 900 replacements would be born to mature ewes. This experiment used additional feed during the weaning-to-breeding period to increase growth rates of the replacements born to ewe lambs. Depending on the individual farm, and the proportion of replacements born to ewe lambs, and the resulting average weaning weights, farmers would need to consider prioritising feeding for the replacements born to ewe lambs; however, farmers may also choose to try and increase growth rates preweaning with additional supplements. A systems analysis is required to determine whether potential increases in genetic gain and efficiency are worth the additional feed costs, and to determine the most feasible proportion of replacements born to ewe lambs.

Differing proportions of the treatment groups were heavy enough to be bred as ewe lambs, driven by the limitation of having to select all lambs born to ewe lambs. In the current experiment, there were 127 days between weaning and the start of ewe lamb breeding, with an average daily liveweight gain of 134 g/d during that period. The minimum recommended breeding weight for ewe lambs is 40 kg [17]. Based on these requirements, ewe lambs would need to have been a minimum of 23 kg at weaning to ensure they were able to be bred at seven months of age. Based on this minimum weaning weight, 100% of the selected M2 ewe lambs, 61% of the L1 ewe lambs, and 22% of the L2 ewe lambs would have been heavy enough for selection at weaning.

At the weaning of the second crop of lambs (December 2019), ewe live weights ranged from 62.4 to 69.9 kg, indicating that they were not likely to be at mature weights. Mature weight is achieved at about 3.5 years of age [11]. Since the ewes are not yet at their mature weight, their production is expected to be less than the national average for number of lambs weaned per ewe bred. The New Zealand national average lambing percentage in 2019 was 127.1% [25], while the ewe groups in this experiment weaned at rates between 105 and 122%. The decrease in live weight prior to the breeding period due to drought conditions will have also impacted reproductive rates, as it is well recognised that a decrease in live weight prior to breeding will decrease ovulation rate [26,27,28,29,30,31,32].

In 2019, there was no difference among the ewe groups for the average lamb weight at weaning, which is consistent with the literature [4,5,7,11]. The M2 ewes produced heavier lambs at birth than the L1 and L2 ewes. This is possibly a maternal constraint on the foetus [29], with the M2 ewes being heavier and larger than the other two groups. The difference in lamb birth weights was only 0.3 kg, and did not affect either the survival rate, or future live weight measurements of the lambs. Therefore, the age of dam and birth rank of the ewe had no effect on the total lamb production during their second lambing.

Lifetime efficiency was measured as the average numbers, weight of lambs weaned per ewe presented for breeding, and the efficiency, as a ratio of litter weight at weaning to ewe breeding weight, of the two lambing opportunities the ewes had. There was unavoidable bias in this analysis, whereby each ewe group had a different proportion of the population presented for breeding during their first year, based on achieving a suitable breeding weight. All M2 ewes—79.3% of L1 ewes and 40.9% of L2 ewes—were presented for breeding as ewe lambs. Consequently, a higher percentage of the breeding records for efficiency were from the second year of breeding for L1 and especially L2 ewes, compared with M2 ewes. With higher rates of production expected from two-year-old ewes than ewe lambs [1], this presents a bias for the analysis. The fixed effect of year was used in the analysis to account for the differences in production between the ewes as one-year-olds vs. two-year-olds. Alternatively, a “zero” production value could have been imposed for the first year of breeding for ewe lambs that were not presented for breeding, but this imposes an even larger bias, as those ewe lambs may have been able to produce but were not given the opportunity. Given the different proportions of each group presented and not presented for breeding, this would impose a large negative bias against the L2 ewes.

While not statistically significant, the L2 ewes produced 2.4 kg less in total litter weaning weight than the M2 ewes in 2019, per ewe bred; however, the lighter live weight of L2 ewes at breeding, and throughout the experimental period, countered this, resulting in very similar efficiency for the two ewe groups. Ewes born as twins to ewe lambs had a lower total litter weaning weight than the other groups and were also lighter at breeding than the ewes born to mature ewes or singles born to ewe lambs, the combined effect showing no difference in efficiency among the groups [11]. The lifetime efficiency values (0.68–0.75; [11]) are higher than the efficiency values for the two combined lambings (0.39–0.40; current experiment), likely due to the differences in the ewe ages during the analysis and the corresponding reproductive rates. The current experiment is the only one to analyse the efficiency including the less-productive year as one-year-olds, and previous work has analysed the ewes during their older years, when they have reached their peak reproductive performance [11]. There was no difference in the litter weights of lambs weaned from singletons either born to ewe lambs or mature ewes, but singletons born to ewe lambs tended to produce heavier litters than singletons born to mature ewes [5]. They also reported that there was generally no difference in ewe live weights after one year of age for singletons born to ewe lambs or mature ewes, and the efficiency of progeny production was also not significantly different among groups. Based on these combined results, if farmers are selecting ewe replacements at weaning, the live weight of the ewe lamb is a much more important criterion to select than the age of the dam or the birth rank of the lamb.

## 5. Conclusions

From 3 to 28 months of age, ewes born to ewe lambs as singletons and twins had lower live weights than ewes born as twins to mature ewes, but they had no difference in body condition scores. Replacements born to ewe lambs had the same lamb production (number and weight of lambs weaned) as replacements born to mature ewes during their first breeding as ewe lambs, provided they were heavy enough at the start of breeding. There were no differences in lamb production during the second year of lambing, or the average lamb production over two years of breeding for those ewes presented for breeding. This suggests that if farmers select ewe lamb replacements from the lamb crop born to ewe lambs based on live weight, they will not sacrifice the production of lambs from their ewes. Ewes born to ewe lambs had the same lamb production, but lighter live weights, possibly making them more efficient, although that was not seen in this experiment. The results reported in this experiment indicate that continued investigation of production of these ewes for their lifetime productivity and longevity is warranted to make more robust recommendations to farmers regarding the selection of lambs born to ewe lambs as flock replacements. Based on the findings presented here, farmers can select replacements out of the lamb crop from their ewe lambs if they are heavy enough to reach breeding weight as ewe lambs on time.

## Figures and Tables

**Table 1:** Percentage of ewe lambs showing oestrous (as an indicator of puberty) prior to the introduction of the ram (Puberty; %), presented for breeding at the start of the breeding period or added to the breeding group for the second half of the breeding period (34 days) for ewe lambs born as twins to mature ewes (M2) or born to ewe lambs as singletons (L1) or as twins (L2) during their first breeding. Values are back-transformed LS means and 95% confidence intervals.

	**M2**	**L1**	**L2**	***p* Value**
n	135	135	88	
Puberty (%) ^1^	21.5 (15.4–29.2) ^b^	15.6 (10.4–22.7) ^ab^	6.8 (3.1–14.4) ^a^	0.0085
Presented at start (%) ^2^	100 *	70.4 (62.1–77.5) ^b^	33.0 (24.0–43.4) ^a^	<0.0001
Presented at all (%) ^3^	100 *	79.3 (71.6–85.3) ^b^	40.9 (31.2–51.4) ^a^	<0.0001

* M2 ewe lambs were all presented for breeding at the start of the breeding period, therefore were not included in the analysis. ^1^ The percentage of ewe lambs with recorded crayon marks from vasectomised rams prior to the introduction of the ram, as an indicator of puberty attainment. ^2^ The percentage of ewe lambs presented for both reproductive cycles of breeding. ^3^ The percentage of ewe lambs presented for breeding (either for both or just the second reproductive cycles). ^a,b^ Means with different superscripts within rows are significantly different at *p* < 0.05.

**Table 2:** Number of ewe lambs diagnosed as pregnant, and number of lambs born (NLB) and weaned (NLW) per ewe lamb presented for breeding in 2018, or per ewe lamb in the entire study population in 2018 and 2019, for ewe lambs born to mature ewes as twins (M2), born to ewe lambs as singletons (L1) or as twins (L2). Values are back-transformed LS means and 95% confidence intervals.

	**M2**	**L1**	**L2**	***p* Value**
**2018**				
n	135	135	88	
Ewe lambs presented for breeding ^1^				
Pregnant (%)	48.9 (40.6–57.3)	52.3 (42.9–61.6)	66.7 (50.0–80.0)	NS
NLB (%)	54.1 (43.0–68.0)	59.8 (46.8–76.4)	75.0 (51.4–109.4)	NS
NLW (%)	45.2 (35.2–58.1)	47.7 (36.2–62.7)	52.8 (33.7–82.7)	NS
All ewe lambs ^2^				
Pregnant (%)	48.9 (40.6–57.3) ^b^	41.5 (33.5–50.0) ^b^	27.3 (19.0–37.5) ^a^	0.0049
NLB (%)	54.1 (43.0–68.0) ^b^	47.4 (37.1–60.6) ^ab^	30.7 (21.0–44.7) ^a^	0.0309
NLW (%)	45.2 (35.2–58.1) ^b^	37.8 (28.7–49.7) ^b^	21.6 (13.8–33.9) ^a^	0.0115
**2019**				
n	133	133	87	
Pregnant (%)	96 (92–99)	94 (88–97)	94 (86–97)	NS
NLB (%)	151 (131–174)	158 (137–181)	151 (126–180)	NS
NLW (%)	122 (105–142)	115 (98–135)	105 (85–129)	NS

^a,b^ Means with different superscripts within rows are significantly different at *p* < 0.05. ^1^ Ewes that were heavy enough (above 39 kg) at either the start of breeding, or the start of the second 17-day period (reproductive cycle) of breeding, over a 34-day (two reproductive cycles) breeding period. ^2^ All ewes in the population, regardless of whether they were exposed to the rams.

**Table 3:** Least squares means (± S.E.M.) of progeny weights at birth, early life (L24 for both 2018 and 2019), and weaning (L95 and L78 for 2018 and 2019, respectively) for ewes born to mature ewes as twins (M2), or born to ewe lambs as singletons (L1) or as twins (L2), and by birth rank of the progeny (singleton, twin, or triplet), during their first (2018) and second (2019) lambings.

	**n**	**Birth (kg)**	**n**	**Early Life (kg)**	**n**	**Weaning (kg)**
**2018**						
Treatment group						
M2	73	4.56 ± 0.10	61	11.55 ± 0.30	60	25.71 ± 0.53
L1	63	4.58 ± 0.10	51	11.08 ± 0.34	50	24.73 ± 0.55
L2	26	4.41 ± 0.17	20	11.22 ± 0.47	19	24.57 ± 0.82
*p* Value		NS		NS		NS
Progeny birth rank						
Singleton	119	4.97 ± 0.08 ^b^	101	12.74 ± 0.23 ^b^	100	27.26 ± 0.41 ^b^
Twin	43	4.07 ± 0.12 ^a^	31	9.83 ± 0.41 ^a^	29	22.75 ± 0.67 ^a^
*p* Value		<0.0001		<0.0001		<0.0001
**2019**						
Treatment group						
M2	188	6.79 ± 0.10 ^b^	159	12.14 ± 0.22	160	25.09 ± 0.36
L1	187	6.49 ± 0.10 ^a^	155	11.97 ± 0.22	153	24.29 ± 0.36
L2	114	6.49 ± 0.12 ^a^	92	11.69 ± 0.27	90	24.45 ± 0.44
*p* Value		0.0136		NS		NS
Progeny birth rank						
Singleton	136	7.92 ± 0.09 ^b^	117	14.63 ± 0.20 ^c^	118	29.11 ± 0.33 ^c^
Twin	326	6.42 ± 0.06 ^a^	268	11.54 ± 0.12 ^b^	264	24.11 ± 0.22 ^b^
Triplet	27	5.43 ± 0.22 ^a^	21	9.76 ± 0.57 ^a^	21	20.61 ± 0.78 ^a^
*p* Value		<0.0001		<0.0001		<0.0001

^a,b^^,c^ Means with different superscripts within columns are significantly different at *p* < 0.05.

**Table 4:** Progeny survival (%) at tagging and weaning (L95 and L78 for 2018 and 2019, respectively) for ewes born to mature ewes as twins (M2), born to ewe lambs as singletons (L1) or as twins (L2), and by birth rank of the progeny (singleton, twin, or triplet), during their first (2018) and second (2019) lambings. Values are back-transformed LS means and 95% confidence intervals.

	**n**	**Survival to Tagging (%)**	**Survival to Weaning (%)**
**2018**			
Treatment group			
M2	73	91.6 (82.0–96.3)	79.3 (67.4–87.7)
L1	63	88.7 (77.8–94.7)	77.1 (64.3–86.3)
L2	26	80.4 (59.8–91.9)	68.5 (47.0–84.2)
*p* Value		NS	NS
Progeny birth rank			
Singleton	119	88.1 (80.4–93.0)	83.0 (74.7–89.0) ^b^
Twin	43	87.2 (72.4–94.6)	65.4 (49.3–78.6) ^a^
*p* Value		NS	<0.05
**2019**			
Treatment group			
M2	188	93.7 (87.7–96.9)	86.6 (79.2–91.6)
L1	187	92.5 (86.2–96.0)	83.3 (75.7–88.9)
L2	114	90.6 (81.7–95.4)	80.5 (70.3–87.9)
*p* Value		NS	NS
Progeny birth rank			
Singleton	136	91.2 (85.0–95.0)	86.9 (80.0–91.7)
Twin	326	92.5 (88.9–95.1)	82.0 (77.1–85.9)
Triplet	27	93.2 (75.7–98.4)	81.5 (61.8–92.3)
*p* Value		NS	NS

^a,b^ Means with different superscripts within column are significantly different at *p* < 0.05.

**Table 5:** The mean number of lambs weaned (NLW), litter weight (kg) and efficiency (ratio of total progeny litter weight to ewe breeding weight) per year for the first two years of breeding for ewes born (Table 2) to ewe lambs as singletons (L1) or as twins (L2). Values for average number of lambs weaned are back-transformed LS means and 95% confidence intervals, and values for litter weight and efficiency are LS means ± S.E.M.

**Ewe Group**	**NLW ^1^**	**Litter Weight (kg) ^2^**	**Efficiency ^3^**
M2	0.75 (0.68–0.82)	21.4 ± 1.0	0.40 ± 0.02
L1	0.74 (0.65–0.83)	20.1 ± 1.1	0.39 ± 0.02
L2	0.67 (0.58–0.78)	19.0 ± 1.6	0.39 ± 0.03
*p* Value	NS	NS	NS

^1^ Average number of lambs weaned each year per ewe presented for breeding. ^2^ Average litter weight at weaning each year per ewe presented for breeding. ^3^ Average efficiency of ewes as a ratio of total progeny litter weight to ewe breeding weight per year for each ewe presented for breeding.

## References

[1] Kenyon P.R., Thompson A.N., Morris S.T. (2014). Breeding ewe lambs successfully to improve lifetime performance. Small Rumin. Res..

[2] Kenyon P.R., Blair H.T. (2014). Foetal programming in sheep—Effects on production. Small Rumin. Res..

[3] Rendell J.M., Robertson A. (1950). Estimation of genetic gain in milk yield by selection in a closed herd of dairy cattle. J. Genet..

[4] Loureiro M.F.P., Pain S.J., Kenyon P.R., Blair H.T. (2016). Reproductive peformance of singleton and twin female offspring born to ewe-lamb dams and mature adult ewes. Proc. N. Z. Soc. Anim. Prod..

[5] Pain S.J., Loureiro M.F.P., Kenyon P.R., Blair H.T. (2015). The effect of dam age on ewe offspring productive performance and efficiency. Proc. N. Z. Soc. Anim. Prod..

[6] Annett R.W., Carson A.F. (2006). Effects of plane of nutrition during the first month of pregnancy on conception rate, foetal development and lamb output of mature and adolescent ewes. Anim. Sci..

[7] Loureiro M.F.P., Pain S.J., Kenyon P.R., Peterson S.W., Blair H.T. (2012). Single female offspring born to primiparous ewe-lambs are lighter than those born to adult multiparous ewes but their reproduction and milk production are unaffected. Anim. Prod. Sci..

[8] Young E.A., Yuan J.V., Everett-Hincks J. (2010). Yearling lambing performance and primary cause of lamb death. Proc. N. Z. Soc. Anim. Prod..

[9] Schreurs N.M., Kenyon P.R., Mulvaney F.J., Morel P.C.H., West D.M., Morris S.T. (2010). Effect of birthweight and birth rank on the survival of single and twin lambs born to ewe lambs. Anim. Prod. Sci..

[10] Pettigrew E.J., Hickson R.E., Blair H.T., Griffiths K.J., Ridler A.L., Morris S.T., Kenyon P.R. (2020). Differences in lamb production between ewe lambs and mature ewes. N. Z. J. Agric. Res..

[11] Pettigrew E.J., Hickson R.E., Morris S.T., Lopez-Villalobos N., Pain S.J., Kenyon P.R., Blair H.T. (2019). The effects of birth rank (single or twin) and dam age on the lifetime productive performance of female dual purpose sheep (Ovis aries) offspring in New Zealand. PLoS ONE..

[12] Cockrem F.R.M. (1979). A review of the influence of liveweight and flushing on fertility made in the context of efficient sheep production. Proc. N. Z. Soc. Anim. Prod..

[13] Coop I.E. (1962). Liveweight-productivity relationships in sheep. N. Z. J. Agric. Res..

[14] Corner-Thomas R.A., Ridler A.L., Morris S.T., Kenyon P.R. (2015). Ewe lamb live weight and body condition scores affect reproductive rates in commercial flocks. N. Z. J. Agric. Res..

[15] Meyer H.H., French R.L. (1979). Hogget liveweight-oestrus relationship among sheep breeds. Proc. N. Z. Soc. Anim. Prod..

[16] Loureiro M.F.P., Paten A.M., Asmad K., Pain S.J., Kenyon P.R., Pomroy W.E., Blair H.T. (2011). BRIEF COMMUNICATION: The effect of dam age and lamb birth rank on the growth rate, faecal egg count and onset of puberty of single and twin female offspring to 12 months of age. Proc. N. Z. Soc. Anim. Prod..

[17] Fact Sheet—Managing Hoggets from Pre-Mating through to Two-Tooths. https://beeflambnz.com/knowledge-hub/PDF/managing-hoggets-pre-mating-through-two-tooths.

[18] Jefferies B.C. (1961). Body condition scoring and its use in management. Tasman. J. Agric..

[19] Radford H.M., Watson R.H. (1960). A crayon and associated harness for the detection of mating under field conditions. Aust. Vet. J..

[20] Whyman D. (1980). Oestrous response of lambs to oestrogen in relation to their fertility at two years of age. N. Z. J. Agric. Res..

[21] O’Connor C.E., Jay N.P., Nicol A.M., Beatson P.R. (1985). Ewe maternal behaviour score and lamb survival. Proc. N. Z. Soc. Anim. Prod..

[22] Griffiths K.J., Ridler A.L., Kenyon P.R. (2017). Longevity and wastage in New Zealand commercial ewe flocks—A significant cost. J. Off. Publ. N. Z. Inst. Prim. Ind. Manag. Inc..

[23] Dýrmundsson Ó.R. (1981). Natural factors affecting puberty and reproductive performance in ewe lambs: A review. Livest. Prod. Sci.

[24] van der Linden D.S., Kenyon P.R., Jenkinson C.M.C., Peterson S.W., Lopez-Villalobos N., Blair H.T. (2007). The effects ewe of size and nutrition during pregnancy on the growth and onset of puberty in female progeny. Proc. N. Z. Soc. Anim. Prod..

[25] Beef + Lamb New Zealand (2019). Lamb Crop Report.

[26] Smith J.F. (1988). Influence of nutrition on ovulation rate in the ewe. Aust. J. Biol. Sci.

[27] Scaramuzzi R.J., Campbell B.K., Downing J.A., Kendall N.R., Khalid M., Munoz-Gutierrez M., Somchit A. (2006). A review of the effects of supplementary nutrition in the ewe on theconcentrations of reproductive and metabolic hormones and the mechanisms that regulate folliculogenesis and ovulation rate. Reprod. Nutr. Dev..

[28] Rattray P.V., Jagusch K.T., Smith J.F., Winn G.W., Maclean K.S. (1981). Effects of genotype, liveweight, pasture type and feeding level on ovulation responses in ewes. Proc. N. Z. Soc. Anim. Prod..

[29] Montgomery G.W., Scott I.C., Johnstone P.D. (1988). Seasonal changes in ovulation rate in Coopworth ewes maintained at different liveweights. Anim. Reprod. Sci.

[30] Morley F.H.W., White D.H., Kenney P.A., Davis I.F. (1978). Predicting ovulation rate from liveweight in ewes. Agric. Syst..

[31] Lassoued N., Rekik M., Mahouachi M., Hamouda M.B. (2004). The effect of nutrition prior to and during mating on ovulation rate, reproductive wastage, and lambing rate in three sheep breeds. Small Rumin. Res..

[32] Ducker M.J., Boyd J.S. (1977). The effect of body size and body condition on the ovulation rate of ewes. Anim. Prod..

